# Linking canopy‐scale mesophyll conductance and phloem sugar δ^13^C using empirical and modelling approaches

**DOI:** 10.1111/nph.17094

**Published:** 2020-12-19

**Authors:** Pauliina Schiestl‐Aalto, Zsofia R. Stangl, Lasse Tarvainen, Göran Wallin, John Marshall, Annikki Mäkelä

**Affiliations:** ^1^ Institute for Atmospheric and Earth System Research (INAR)/Forest Sciences Helsinki 00014 Finland; ^2^ Department of Forest Ecology and Management SLU Umeå 901 83 Sweden; ^3^ Department of Biological and Environmental Sciences University of Gothenburg Gothenburg 405 30 Sweden

**Keywords:** ^13^C discrimination, dynamic model, mesophyll conductance, photosynthesis, *Pinus sylvestris*, stable carbon isotopes

## Abstract

- Interpreting phloem carbohydrate or xylem tissue carbon isotopic composition as measures of water‐use efficiency or past tree productivity requires in‐depth knowledge of the factors altering the isotopic composition within the pathway from ambient air to phloem contents and tree ring. One of least understood of these factors is mesophyll conductance (*g*_m_).
- We formulated a dynamic model describing the leaf photosynthetic pathway including seven alternative *g*_m_ descriptions and a simple transport of sugars from foliage down the trunk. We parameterised the model for a boreal Scots pine stand and compared simulated *g*_m_ responses with weather variations. We further compared the simulated δ^13^C of new photosynthates among the different *g*_m_ descriptions and against measured phloem sugar δ^13^C.
- Simulated *g*_m_ estimates of the seven descriptions varied according to weather conditions, resulting in varying estimates of phloem δ^13^C during cold/moist and warm/dry periods. The model succeeded in predicting a drought response and a postdrought release in phloem sugar δ^13^C indicating suitability of the model for inverse prediction of leaf processes from phloem isotopic composition.
- We suggest short‐interval phloem sampling during and after extreme weather conditions to distinguish between mesophyll conductance drivers for future model development.

## Introduction

Stable carbon isotopic composition (δ^13^C) of tree rings has been used to inform us about past climate in paleo‐ecological records since the 1970s (Libby *et al*., 1976; Wilson & Grinsted, 1977; Zeng *et al*., 2017). After working out how wood isotopic composition is related to variation in the intrinsic water‐use efficiency of photosynthesis, many papers have been published describing variation in iWUE (e.g. Francey & Farquhar, 1982; McCarroll & Loader, 2004; Voelker *et al*., 2019) and with the objective of quantifying past tree productivity (Rascher *et al*., 2010; Schollaen *et al*., 2013). The basis for these analyses was laid down by the steady‐state model of photosynthetic carbon isotope fractionation by Farquhar *et al*. (1980, 1989) and its further developments (e.g. Lloyd & Farguhar, 1994). A straightforward application of these models provides a simple ‘inverse’ method for estimating leaf processes from xylem isotopic ratios (McCarroll & Loader, 2004). Although the simple model is often sufficient, there are instances in which more detailed descriptions are needed (Cernusak *et al*., 2013). Of particular interest in the last decade has been the influence of mesophyll conductance (*g*_m_) (Flexas *et al*., 2012) and a set of postphotosynthetic isotopic fractionation processes (Francey *et al*., 1985; Gessler *et al*., 2014; Rinne *et al*., 2015). A mechanistic understanding of these processes would lead to much more complex models than the early ‘inverse’ isotope models (Danis *et al*., 2012). An important step towards that is to quantify the additional influences on the isotopic signal (Danis *et al*., 2012; Cernusak *et al*., 2013) after which these more comprehensive models could be solved by means of modern data‐model assimilation methods, such as Bayesian analysis (Van Oijen, 2017).

The first step of the isotopic path occurs when carbon dioxide enters the intercellular airspaces (Lloyd & Farguhar, 1994) controlled by stomatal conductance (*g*_s_). From the intercellular airspaces to chloroplasts, CO_2_ encounters a series of resistances that aggregate to mesophyll resistance or, inversely, mesophyll conductance (Evans *et al*., 2009; Pons *et al*., 2009). *g*_m_ was for a long time not explicitly considered in photosynthesis models. Recent evidence shows, however, that mesophyll conductance may strongly limit the carbon flux to chloroplasts (Pons *et al*., 2009; Flexas *et al*., 2012; Sun *et al*., 2014; Ogée *et al*., 2018) and affect the isotopic signal. Many studies have demonstrated a response of *g*_m_ to environmental and internal controls (Stangl *et al*., 2019; Knauer *et al*., 2020), such as light (Campany *et al*., 2016), temperature (Evans & von Caemmerer, 2013), nutrients (Bown *et al*., 2009) and CO_2_ concentration in intercellular airspaces (*C*_i_) or chloroplast (*C*_c_) (Flexas *et al*., 2007; Tazoe *et al*., 2011). However, few models using dynamic *g*_m_ have been presented (Sun *et al*., 2014; Dewar *et al*., 2018). Instead, mesophyll conductance has mostly been expressed either as a constant or as a constant ratio to *g*_s_ (Flexas *et al*., 2008). At the same time, *g*_m_ has been identified as one of the most important missing factors from terrestrial biosphere models and land surface models (Rogers *et al*., 2017; Knauer *et al*., 2020). Estimates of *g*_m_ vary between tree species (Warren, 2008; Flexas *et al*., 2012). Our analysis was conducted on Scots pine (*Pinus sylvestris*). For *Pinus* species *g*_m_ values 0.04–0.4 mol m^−2^ s^−1^ have been reported (De Lucia *et al*., 2003; Flexas *et al*., 2008; Maseyk *et al*., 2011; Veromann‐Jürgenson *et al*., 2017; Stangl *et al*., 2019).

In the chloroplasts, carboxylation produces sugars in reactions with specific isotopic fractionation characteristics (Farquhar *et al*., 1982; McNevin *et al*., 2006). A part of these sugars is loaded to the phloem and transported to other tree organs (Desalme *et al*., 2017). Rascher *et al*. (2010) observed that phloem sap δ^13^C of mature maritime pines correlated with environmental factors with a 4‐d time lag. This implies that phloem sap δ^13^C would follow the δ^13^C of whole canopy assimilates (Ubierna & Marshall, 2011), except for a time lag caused by a finite phloem transport rate, and thus could be used as an indicator of leaf processes. After photosynthesis, however, the isotopic signal may be weakened by the mixing of the newly synthesised sugars with those stored earlier (Offermann *et al*., 2011) or additional postphotosynthetic fractionation for example in sugar compound conversions, structural growth or respiration (Tcherkez *et al*., 2004; Badeck *et al*., 2005; Priault *et al*., 2009; Merchant *et al*., 2011; Rinne *et al*., 2015). These fractionation effects may need to be quantified if phloem sap or xylem tissue δ^13^C is used for precise estimates of photosynthate δ^13^C.

Under the steady‐state assumption of the seminal modelling work of Farquhar *et al*. (1980, 1989), carbon flux into the leaf equals net photosynthesis (*A*_net_) (von Caemmerer, 2013) and the δ^13^C of new photosynthates can be derived from the δ^13^C of the CO_2_ flux into the leaf. During high‐flux conditions, when the ratio of photosynthetic rate to respiratory rate is large, this derivation of δ^13^C of new photosynthates is most probably accurate. However, misinterpretation of the results is possible during mornings and evenings when the photosynthetic rate is low compared with the respiratory rate (Busch *et al*., 2020). High‐resolution measurements of photosynthesis and discrimination would be required to test the effects of different model assumptions on the accuracy of the ^13^C discrimination prediction. Such data are rarely available, as these measurements are technically challenging under field conditions (Stangl *et al*., 2019). However, model inspection can help to quantify the conditions in which neglecting the effects of these factors is significant.

This study was carried out with the ultimate objective of developing a tool to estimate tree WUE from a relevant set of weather input variables. For this, we evaluated different hypotheses on mesophyll conductance that could be used as a component of an inverse model for estimating leaf fluxes from phloem isotopic composition. We first formulated a dynamic model of isotopic fractionation in the leaf, then combined this with a simple description of transport of sugars down the phloem. The leaf model is essentially a dynamic version of the steady‐state model presented by Farquhar *et al*. (1982, 1989). It describes the photosynthesis of ^12^C and ^13^C, taking into account fractionation in fluxes through stomata and mesophyll, RuBisCo activity, as well as mitochondrial respiration and photorespiration. We formulated and compared seven mesophyll conductance descriptions that are based on published models of *g*_m_ (Sun *et al*., 2014; Dewar *et al*., 2018) and recent data from our measurement site (Stangl *et al*., 2019). We used continuous gas‐exchange measurements conducted at Rosinedal experimental forest in northern Sweden in 2017 to parameterise the model and concurrent carbon isotope measurements to compare modelled *g*_m_ with measurement‐based estimates of *g*_m_. Furthermore, the temporal pattern of phloem δ^13^C was measured at the site in 2018. On the basis of the results we discuss the potential of using the approach as a tool for inverse modelling of gas‐exchange or water‐use efficiency from phloem sap δ^13^C and environmental conditions, as well as to consider the possible benefits of the dynamic approach taken in the leaf model.

The main aims were:

1. To compare the seven different *g*_m_ descriptions, and to analyse their implications for the predicted δ^13^C of photosynthesised sugars.
2. To study the environmental sensitivity of δ^13^C of phloem sugars under the different *g*_m_ descriptions, and to test the respective predictions against phloem δ^13^C data during the summer drought of 2018.
3. To study the diurnal patterns of δ^13^C within the photosynthetic pathway.

## Materials and Methods

### Study site

Rosinedalsheden is a 100‐yr‐old Scots pine experimental forest located in northern Sweden (64°10′N, 19°45′E) with a cool boreal climate. The mean temperature of the summer months was 12.4°C and mean monthly precipitation 67.9 mm (average of years 2003–2017). The site had low‐fertile fine sandy soil with a 2–5 cm organic layer on top (Hasselquist *et al*., 2012). The average tree height was 18.6 m and leaf area index 2.7 m^2^ m^−2^ (Lim *et al*., 2015).

### Gas‐exchange and δ^13^C measurements

CO_2_ and H_2_O exchange was continuously measured during the 2017 growing season on 1‐yr‐old attached shoots in the upper canopy of four pine trees. The tree canopies were accessed with 16 m high scaffolding towers built inside the stand. Shoots were enclosed in 330 ml transparent cuvettes tracking the ambient temperature by means of Peltier heat exchangers (Tarvainen *et al*, 2016). The difference between cuvette and ambient temperature was on average + 0.1°C and < ± 0.5°C for 97% of the time. Photosynthetic photon flux density (PPFD) was measured next to each cuvette with a leveled and cosine‐corrected quantum sensor (PAR‐1(M); PP Systems, Hitchin, Herts, UK). During the measurements ambient air was continuously drawn into the shoot cuvettes and adjacent empty reference cuvettes of the same volume. From there, heated and insulated polyethene tubing connected the cuvettes to a multichannel gas‐exchange system equipped with infrared gas analysers (IRGA, CIRAS‐1, PP systems) running in open mode (Wallin *et al*., 2001). The airflow through the entire system was generated using diaphragm pumps. Vapour pressure deficit was calculated according to Buck (1981). Ambient VPD was, on average, 0.2 kPa higher than inside the cuvettes. In addition, δ^13^C in the shoot cuvettes was measured using a cavity ring‐down spectrophotometer (CRDS; G2131‐i, Picarro Inc., CA, USA) connected to the gas‐exchange system. The CRDS analyser was calibrated once per week with two reference gases (411 µmol mol^−1^ CO_2_, δ^13^C = −32.4‰ and 1606 µmol mol^−1^ CO_2_, δ^13^C = −4.1‰).

Net photosynthesis and stomatal conductance were calculated for a sequence of days with high data quality from the gas‐exchange data following Farquhar *et al*. (1980) and the δ^13^C of net photosynthesis was determined during the summer 2017. More details of the measurement system and calculations are presented in Stangl *et al*. (2019).

### Phloem sugar isotopes

Phloem contents were collected from three trees within the same stand at 2–4‐wk intervals between late‐June and early‐October in 2018. Samples were taken with a hole punch (∅ =10 mm) at 1.3 m height. The phloem discs were put into 1.5 ml de‐ionised water for 12–17 h at 10°C to extract the phloem contents. The tissue was removed and the samples were dried in a centrifuge connected to a cold‐trap. The isotopic composition of the phloem content was analysed by GB‐IRMS (Gasbench II – Isotope Ratio Mass Spectrometer; Thermo Fisher Scientific, Bremen, Germany) calibrated against IAEA‐co‐9 and NBS 19 standards (SLU Stable Isotope Laboratory, Umeå, Sweden).

### Environmental variables

**Fig. 1:**
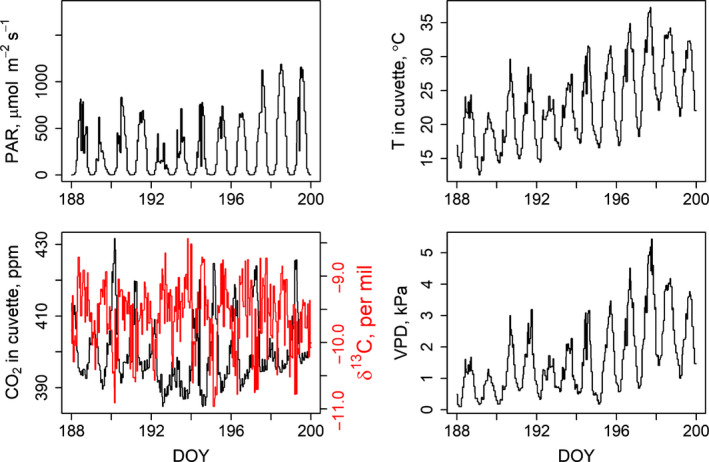
(a) Photosynthetically active radiation, (b) temperature, (c) CO_2_ concentration (black) and its isotopic composition (red) in the cuvette and (d) vapour pressure deficit during the study period. DOY, day of year.

Half‐hourly air temperature and relative humidity were measured at 1.5 m height with a HC2‐S3 probe (Rotronic AG, Bassersdorf, Switzerland) installed in a ventilated radiation shield (In Situ, Ockelbo, Sweden). Half‐hourly above‐canopy PPFD was measured with a Li‐190SA PPFD‐sensor (Li‐Cor Biosciences, Lincoln, NE, USA) (Fig. 1).

### The model

#### Leaf carbon pools and fluxes

**Fig. 2:**
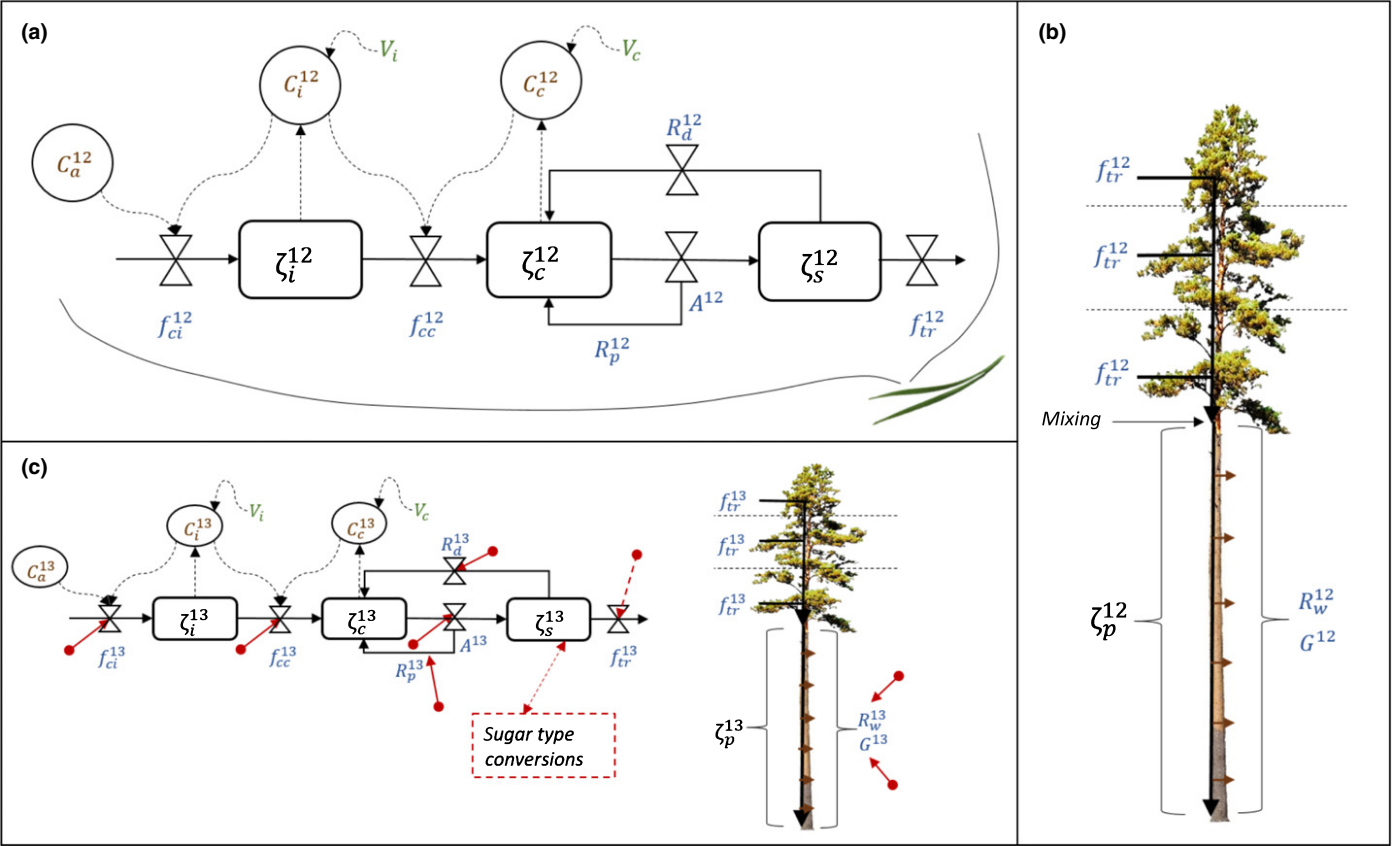
Model structure. Panels (a, b) represent the leaf model and the following transport of carbon downwards via phloem, respectively, for ^12^C whereas (c) shows the same for ^13^C with red arrows indicating processes that include isotopic discrimination, that is the processes in which the isotopic signal is altered. Dashed red arrows show processes with potential additional discrimination. *ζ_I_* are the pools of ^12^C and ^13^C, that is the state variables of the model and they are expressed as carbon per leaf area (mol m^−2^) in intercellular airspaces, chloroplasts or leaf nonstructural sugar pool (*I = i*, *c*, *s*, respectively, leaf model) or nonstructural carbon in phloem per metre trunk (mol m^‐1^, *I = p*, trunk model). *C_I_* (brown colour) denotes the carbon concentrations in the corresponding pools that can be calculated with pool size *ζ_I_* and pool volume *V_I_*. *C_a_* is carbon concentration in ambient air. Blue colours indicate fluxes into the pools, between the pools or out of the pools (mol m^−2^ s^−1^). *f*_ci_ and *f*_cc_ are the fluxes to intercellular airspaces and chloroplasts, respectively, *A* is photosynthetic rate, *R_d_* and *R_p_* mitochondrial and photorespiration, respectively, *f_tr_* is export of sugar from leaf to phloem and *R_w_* and *G* trunk respiration and growth, respectively.

The state variables of the model are pools of carbon in leaf intercellular airspaces ($\zeta_{i}^{j}$) in chloroplasts ($\zeta_{c}^{j}$) and leaf sugar pool ($\zeta_{s}^{j}$), expressed per leaf area (mol m^−2^) (Fig. 2). *j* denotes isotopes ^12^C or ^13^C and ‘sugar pool’ refers to total nonstructural carbohydrates. The pools can be converted to CO_2_ concentrations, $C_{I}$ (mol mol^−1^):

(Eqn 1) $$C_{I}=\zeta_{I}RT/V_{I},$$

where *V*_I_ is the volume of the intercellular airspaces (*V*_i_) or chloroplasts (*V*_c_) per leaf area (m^3^ m^−2^).

The rates of change of the state variables (mol m^−2^ s^−1^), are:

(Eqn 2) $$\frac{d\zeta_{i}^{j}}{dt}=f_{\mathrm{ci}}^{j}‐f_{\mathrm{cc}}^{j}$$

(Eqn 3) $$\frac{d\zeta_{c}^{j}}{dt}=f_{\mathrm{cc}}^{j}‐A_{c}^{j}+R_{d}^{j}+R_{P}^{j}$$

(Eqn 4) $$\frac{d\zeta_{s}^{j}}{dt}=A^{j}‐R_{d}^{j}‐f_{\mathrm{tr}}^{j}$$

where $f_{\mathrm{ci}}^{j}$ is the carbon flux into the leaf through stomata, $f_{\mathrm{cc}}^{j}$ the carbon flux into the chloroplasts through the mesophyll, *A^j^* carboxylation rate, $R_{d}^{j}$ and $R_{P}^{j}$ the rates of mitochondrial respiration and photorespiration, respectively and *f*_tr_ is the rate of carbon transport from the leaves. Here, respired CO_2_ is released into $\zeta_{c}^{j}$. In reality CO_2_ is released in between *ζ*_c_ and *ζ*_i_ (Tholen *et al*., 2012; Ubierna *et al*., 2019). Thus, we tested the effect of the contrary assumption of CO_2_ being released into $\zeta_{i}^{j}$ (see Section ‘Deriving δ^13^C of photosynthates from CO_2_ concentration inside a cuvette’).

Following Farquhar *et al*. (1989), the fluxes (mol m^−2^ s^−1^) are:

(Eqn 5) $$f_{\mathrm{ci}}^{12}=g_{s}\left(C_{a}^{12}‐C_{i}^{12}\right)$$

(Eqn 6) $$f_{\mathrm{ci}}^{13}=\frac{g_{s}}{1+a_{s}}\left(C_{a}^{13}‐C_{i}^{13}\right)$$

(Eqn 7) $$f_{\mathrm{cc}}^{12}=g_{m}\left(C_{i}^{12}‐C_{c}^{12}\right)$$

(Eqn 8) $$f_{\mathrm{cc}}^{13}=\frac{g_{m}}{1+a_{m}}\left(C_{i}^{13}‐C_{c}^{13}\right)$$

where *g*_s_ and *g*_m_ (mol m^−2^ s^−1^) are stomatal conductance to CO_2_ and mesophyll conductance, respectively, *a*_s_ and *a*_m_ are ^13^C/^12^C fractionation related to *g*_s_ and *g*_m_, respectively, and *C*_a_, *C*_i_ and *C*_c_ are the mole fractions of CO_2_ in ambient air, leaf cellular airspaces and chloroplasts, respectively.

The rate at which carbon is taken to Calvin cycle is determined by a ‘bisubstrate model’ (Thornley & Johnson, 1990; Dewar *et al*., 2018) amended with a seasonality effect (Hari & Mäkelä, 2003; Mäkelä *et al*., 2004):

(Eqn 9) $$A_{c}^{12}\left(t\right)=f_{T}^{A}\left(t\right)f_{S}^{A}\left(t\right)\frac{\alpha I\left(t\right)C_{c}^{12}\left(t\right)}{C_{c}^{12}\left(t\right)+\alpha I\left(t\right)r_{x0}{+\Gamma }^{*}\left(t\right)}$$

where $f_{T}^{A}\left(t\right)$ is direct temperature effect on photosynthetic rate, $f_{S}^{A}\left(t\right)$ a delayed temperature effect describing seasonal acclimation, *I*(*t*) PAR (mol m^−2^ s^−1^), $\Gamma^{*}\left(t\right)$ the light compensation point of photosynthesis, α maximum quantum yield and *r_x0_* carboxylation resistance coefficient:

(Eqn 10) $$A_{c}^{13}\left(t\right)=R_{c}\left(t\right)\frac{A_{c}^{12}\left(t\right)}{1+b}$$

where *R*_c_ is the isotopic ratio of carbon in chloroplasts ($\frac{\zeta_{c}^{13}}{\zeta_{c}^{12}}$) and *b* discrimination related to RuBisCo activity (Farquhar *et al*., 1989). During this process, part of the carbon is released via photorespiration back inside the leaf (Busch, 2013):

(Eqn 11) $$R_{P}^{12}\left(t\right)=\frac{A_{c}^{12}\left(t\right)}{C_{c}^{12}\left(t\right)/\Gamma^{*}\left(t\right)}$$

(Eqn 12) $$R_{P}^{13}\left(t\right)=\frac{A_{c}^{13}\left(t\right)}{A_{c}^{12}\left(t\right)}\frac{R_{P}^{12}\left(t\right)}{\left(1+f\right)}$$

where *f* is a discrimination parameter (Lloyd & Farguhar, 1994). Thus, final carbon bound in photosynthesis (mol m^−2^ s^−1^) to new sugars is:

(Eqn 13) $$A^{j}\left(t\right)=A_{c}^{j}\left(t\right)‐R_{P}^{j}\left(t\right)$$

*A^j^* enters the pool of photosynthesised carbon $\zeta_{s}^{j}$ that is either stored in leaves, transported to other tree parts or released in mitochondrial respiration. The retention time of sugars in $\zeta_{s}^{j}$ is described with time constant (*τ*_R_) with its inverse describing the rate of sugar export from the leaf:

(Eqn 14) $$f_{\mathrm{tr}}^{12}=\frac{\zeta_{s}^{12}}{\tau_{R}}$$

(Eqn 15) $$f_{\mathrm{tr}}^{13}=\frac{\zeta_{s}^{13}}{{(1+h_{\mathrm{tr}})\tau }_{R}}$$

where *h*_tr_ is ^13^C discrimination parameter related to sugar conversion and transport.

#### Mesophyll conductance

Following previously suggested equations or hypotheses about factors determining mesophyll conductance, we formulated seven *descriptions* of *g*_m_ (mol m^−2^ s^−1^) (Table 1); assuming a connection between *g*_m_ and photosynthetic rate (*descriptions 1*, *2*, and *5*), estimating *g*_m_ solely from environmental parameters (*descriptions 3* and *4*), or assuming constant *g*_m_ (*descriptions 6* and *7*).

**Table 1:** Mesophyll conductance (mol m^−2^ s^−1^) equations.

Description	Equation	Affecting factors	References
1	$g_{m}\left(t\right)=g_{m}^{0}+\frac{{a_{2}A}^{12}\left(t\right)}{C_{c}\left(t\right)‐\Gamma^{*}}r_{W}\left(t\right)$	Photosynthetic rate, *C*_c_, water stress	Dewar *et al*. (2018)
2	$g_{m}\left(t\right)=g_{m}^{0}+\frac{{a_{2}A}^{12}\left(t\right)}{C_{c}\left(t\right)‐\Gamma^{*}\left(t\right)}$	Photosynthetic rate, *C*_c_	Dewar *et al*. (2018)
3	$g_{m}\left(t\right)=g_{m}^{0}+g_{m}^{25}r_{T}\left(t\right)r_{I}^{l}$	Temperature, light environment	Sun *et al*. (2014)
4	$g_{m}\left(t\right)=g_{m}^{0}+g_{m}^{25}r_{T}\left(t\right)r_{I}^{l}r_{W}\left(t\right)$	Temperature, light environment, water stress	Sun *et al*. (2014)
5	$g_{m}\left(t\right)=g_{m}^{0}+\frac{{a_{2}A}^{12}\left(t\right)}{C_{c}\left(t\right)‐\Gamma^{*}\left(t\right)}r_{T}\left(t\right)$	Photosynthetic rate, *C*_c_, temperature	Dewar *et al*. (2018), Sun *et al*. (2014)
6	$g_{m}=g_{m}^{c,l}$	Constant *g*_m_	Stangl *et al*. (2019)
7	$g_{m}=g_{m}^{c,h}$	Constant *g*_m_	Approximating infinite *g*_m_

$g_{m}^{0}$ is the minimum mesophyll conductance, *a*_2_ is a parameter, *A* ^12^ is photosynthetic rate (mol m^−2^ s^−1^), *C*_c_ is the CO_2_ concentration in chloroplasts, $g_{m}^{25}$ is mesophyll conductance at 25°C, *r*_W_, *r*_T_ and *r*_I_ represent the effects of water, temperature and light environment (ϵ[0, 1], unitless), respectively (Supporting Information Eqns S1.9, S1.13 and S1.14). The value of *r*_I_ varies over canopy layers but is constant over time.

#### Other variables

*g*_s_ was described with Ball–Berry–Leuning function (Leuning, 1995). *R*_d_ was calculated following Launiainen *et al*. (2015) and its isotopic discrimination with the δ^13^C of leaf sugar pool and a discrimination parameter *e*. Equations related to these variables as well as light compensation point of photosynthesis, direct and lagged effect of temperature on photosynthetic rate and effects of water stress and temperature on mesophyll conductance are presented in Supporting Information Methods S1.

#### Tree canopy structure

The tree canopy was vertically divided into three parts. Previous observations show that PAR decreases by 41% and 65% to the middle and lowest layers, respectively (Tarvainen *et al*., 2016). According to the observation of declining stomatal conductance with canopy depth (G. Wallin, unpublished data), we reduced the value of parameter *a*_1_ by 15% to the second and 30% to the lowest canopy layer to produce the observed increase in the *C*_i_ : *C*_a_ ratio. This parameterisation can be adjusted according to data availability in future applications of the model.

#### Transport of sugars from leaves to phloem

We assumed that in the middle canopy layer, on average, 60% of the photosynthates were transported to the stem and roots and 40% used for branch maintenance and growth (Schiestl‐Aalto *et al*., 2019). We further assumed that the proportions of transported sugars from the other layers were related to the ratios of photosynthetic rates between the layers. Transport and storage of recent assimilates require conversion of glucose to other soluble sugars or starch. The isotopic effect of these conversions can be expressed in analogy to other fluxes (see e.g. Eqn 12). In this first model version we, however, assumed no discrimination related to conversion.

The recent assimilates were mixed with the sugar pool of leaves and at the canopy bottom, the sugar transported from the canopy layers was mixed with the existing pool of phloem sugars. The sizes of the sugar pools were set to 6.7 gC m^−2^ leaf and 27 gC m^−1^ trunk for leaves and phloem, respectively, based on the measurements by Schiestl‐Aalto *et al*. (2019) conducted in a boreal Scots pine stand.

When carbon is drawn out of the phloem for use, there may be discrimination related to either respiration or structural growth, causing the rest of the phloem sugars to be either depleted or enriched. Thus, a vertical gradient would form in the δ^13^C of the phloem sugars. Rascher *et al*. (2010) observed a depletion of δ^13^C of −0.8 ‰ from canopy to the trunk for *Pinus pinaster*. By contrast, Gessler *et al*. (2009) found a 1.5‰ enrichment from leaf soluble matter to phloem content in the trunk in Scots pine trees. We set the discrimination parameter related to trunk maintenance respiration to −6‰ (Ghashghaie *et al*., 2003) and assumed that $20\times \left(\frac{T(t)}{25}\right)\%$, where *T*(*t*) is ambient temperature, of the transported carbon was respired, which led to a reasonable yearly proportion of stem respiration (Schiestl‐Aalto *et al*., 2015). Lacking sufficient knowledge, we set the discrimination parameter related to structural growth to zero. Furthermore, we ignored the possible effects of corticular photosynthesis in the stem tissues (Tarvainen *et al*., 2018) on the trunk δ^13^C but were able to include that in future model versions.

The rate of phloem transport was assumed constant 15 cm h^−1^ (Högberg *et al*., 2008). This caused a time lag between the δ^13^C of new photosynthates and trunk phloem sugars. A more detailed phloem transport submodel can be adopted in further versions of the model.

#### Simulations

$C_{i0}^{12}$ was set to 300 µmol mol^−1^ and $C_{c0}^{12}$ to 200 µmol mol^−1^. We assumed that the initial δ^13^C of *ζ*_i_ and *ζ*_c_ equal the δ^13^C of ambient air. Initial $\zeta_{s}^{12}$ pool was set to equal the later average pool size and isotopic composition set to −26‰ to produce reasonable respiration values right from the beginning of the simulation.

Environmental data measured with 15–30 min interval were linearly interpolated to form an input data series with a 15 min time step. The model simulation used variable time steps. As the changes of rates of carbon fluxes were caused by changes in environmental variables, the essential dynamic of the model occurred at the time of change of the drivers and then settled down to a steady state. In the beginning of each 15 min time step with possible changes in the drivers, the simulation used a time step of 1/250 to 1/4 s, depending on the rates of *A*_c_, *R*_d_ and *R*_p_ until the system reached steady state, that is when the rates of change of the state variables *ζ*_i_ and *ζ*_c_ dropped below threshold *r*_ss_ (1 × 10^−20^ mol mol^−1^). After reaching the steady state, the simulation moved to the end of the ongoing 15‐min period.

#### Parameter estimation

The most important parameters related to photosynthesis and *g*_s_, the slope of the Ball–Berry–Leuning function (*a*_1_), quantum yield (*α*) and carboxylation resistance (*r_x0_*), were estimated separately for each *g*_m_ *description* by fitting the simulated leaf carbon influx (*f*_ci_) and stomatal conductance (*g*_s_) to cuvette measurements with R‐software nonlinear least squares function (R Core Team, 2017). The fitting was carried out for days of the year 172–193 of 2017 with high quality *A*_net_ and *g*_s_ measurements. Measurements were conducted on upper canopy shoots. As the vertical variation in the photosynthetic parameters in the studied trees is small (Tarvainen *et al*., 2016), we used the estimated parameters for all canopy layers. Other parameters were taken from previous measurements conducted at the site or from the published literature (Table 2).

**Table 2:** Model parameters.

Parameter	Value	Unit	Equation	Parameter explanation
*p*_norm_	1013	hPa		Atmospheric pressure
*a*_1_	4.2	–	S1.1	*g*_s_ parameter
*a*_2_	6.0	–	Table 1	*g*_m_ parameter
*a_m_*	1.8 × 10^−3^	–	8	Discrimination parameter
*a_s_*	4.4 × 10^−3^	–	6	Discrimination parameter
*b*	29 × 10^−3^	–	10	Discrimination parameter
*C*_i0_	300 × 10^−6^	mol mol^−1^		Initial CO_2_ concentration
*C*_c0_	200 × 10^−6^	mol mol^−1^		Initial CO_2_ concentration
*D*_0_	2	kPa	S1.1	Threshold VPD
*d*_1_	0.08	C^−1^	S1.6	Parameter of direct temperature effect
*d*_2_	−5.0	C	S1.6	Parameter of direct temperature effect
*e*	−6	–	S1.3	Discrimination parameter
*f*	11 × 10^−3^	–	12	Discrimination parameter
$g_{1}$	36.9 × 10^−6^	–	S1.5	Light compensation point parameter
$g_{2}$	1.88 × 10^−6^	K^−1^	S1.5	Light compensation point parameter
$g_{3}$	0.036 × 10^−6^	K^−1^	S1.5	Light compensation point parameter
$g_{s}^{0}$	0.003	mol m^−2^ s^−1^	S1.1	Minimum *g*_s_
$g_{m}^{0}$	0.003	mol m^−2^ s^−1^	Table 1	Minimum *g*_m_
$g_{m}^{25}$	0.5	mol m^−2^ s^−1^	Table 1	*g*_m_ at 25°C
$g_{m}^{c,l}$	0.4	mol m^−2^ s^−1^	Table 1	constant *g*_m_
$g_{m}^{c,h}$	0.9	mol m^−2^ s^−1^	Table 1	constant *g*_m_
*h*_tr_	0	–	15	Discrimination parameter
$i_{I}^{1}$	0.96	–	S1.14	Parameter of light effect on *g*_m_
$i_{I}^{2}$	0.89	–	S1.14	Parameter of light effect on *g*_m_
$i_{I}^{3}$	0.83	–	S1.14	Parameter of light effect on *g*_m_
*I*_RT_	50 × 10^−6^	mol m^−2^ s^−1^	19	Threshold PAR
LAI	2.7	m^2^ m^−2^		Leaf area index
*M*_CO2_	44	g mol^−1^		CO_2_ molar mass
*M*_C_	12	g mol^−1^		C molar mass
*p*_1_	20	–	S1.13	Parameter of T effect on *g*_m_
*p*_2_	49.6 × 10^3^	Pa m^3^ mol^−1^	S1.13	Parameter of T effect on *g*_m_
*p*_3_	1.4 × 10^3^	Pa m^3^ mol^−1^ K^−1^	S1.13	Parameter of T effect on *g*_m_
*p*_4_	437.4 × 10^3^	Pa m^3^ mol^−1^	S1.13	Parameter of T effect on *g*_m_
*p*_D_	5.0	kPa^−1^	S1.10	Parameter of VPD effect on *g*_m_
*p*_S_	2.5	–	S1.11	Parameter of soil moisture effect on *g*_m_
*R*	8.314	Pa m^3^ mol^−1^ K^−1^	1, S1.2	Gas constant
*r*_1_	32 500	K^−1^	S1.2	Mitochondrial respiration parameter
*r*_2_	298	mol Pa^−1^ m^−3^	S1.2	Mitochondrial respiration parameter
*R_d_*_,_ *_25_*	9 × 10^−6^	mol m^−2^ s^−1^	S1.2	Mitochondrial respiration at 25 °C
*r*_ss_	1 × 10^−20^	mol mol^−1^	2.5.6	Steady‐state threshold
*r_x0_*	5.9	mol^−1^ m^2^ s	9	Carboxylation resistance
*S*_max_	17.3	C	S1.7	Parameter of lagged temperature effect
*T*_N25_	298.15	K	S1.2, S1.5	Temperature, 25 °C
*V_i_*	1 × 10^−4^	m^3^ m^−2^	1	Intercellular airspace volume
*V_c_*	3 × 10^−4^	m^3^ m^−2^	1	Mesophyll volume
*α*	0.14	mol mol^−1^	9	Maximum quantum yield
*θ*_WP_	0.059	m^3^ m^−3^	S1.12	Wilting point
*θ*_FC_	0.222	m^3^ m^−3^	S1.12	Field capacity
*τ*_R_	1	Days	14, 15	Time constant of respiration substrate
*τ*_S_	16.1	Day	S1.8	Time constant of lagged temperature effect

In *g*_m_ *descriptions 2*, *3*, *5* and *6* we set parameters *a*_2_ and $g_{m}^{25}$ so that modelled average midday *g*_m_ corresponded with the measurements conducted at the site (Stangl *et al*., 2019). Values of *a*_2_ and $g_{m}^{25}$ of *descriptions 2* and *3* were the adopted to *descriptions 1* and *4*, that further included water‐stress reduction (Table 1).

We tested the sensitivity of the model to parameters that were most uncertain and yet important for interpreting the results: *e*, *f*, *τ_R_*, *d*_1_ and *d*_2_. Furthermore, we tested the sensitivity of the model results on varying parameter *α* while keeping other parameters as estimated. Parameter estimation is explained in detail in Methods S2.

### Analyses

#### Effect of different *g*_m_ models on predicted *g*_m_ and the isotopic composition of assimilated sugars

We studied the effect of different mesophyll conductance *descriptions* on the within‐day and among‐days variations of predicted mesophyll conductance. Furthermore, we studied how these differences were reflected in the isotopic composition of assimilated sugars.

#### Effect of different *g*_m_ models on predicted phloem sugar δ^13^C

We simulated the isotopic composition of phloem sugars at breast height for year 2018 with the seven *g*_m_ *descriptions* using photosynthetic parameters estimated for year 2017 and compared the simulated phloem δ^13^C values with the measured values. We tested the environmental sensitivity of the isotopic signature of phloem sugars under different *g*_m_ *descriptions* by running the model under hypothetical weather inputs, including temperature, RH and light (Fig. S1). The objective was to identify the input combinations that could tease out critical differences in the output phloem isotopes and thus best inform us about the drivers of mesophyll conductance.

#### Deriving δ^13^C of photosynthates from CO_2_ concentration inside a cuvette

In the cuvette measurements, the difference between CO_2_ concentration inside and outside the cuvette implies the rate of carbon flux from the cuvette into, or out of, the leaf, that is *f*_ci_ (Fig. 2; Eqns 5, 6) and, following the steady‐state assumption, is interpreted as net photosynthesis (${f_{\mathrm{ci}}=A}_{\mathrm{net}}=A‐R_{d}‐R_{p}$). When photosynthetic rate (*A*) is high, *A* is the dominating flux over *R*_d_ and *R*_p_ and thus roughly equals *f*_ci_ and *f*_cc_, the fluxes of CO_2_ through stomata and mesophyll, respectively. Therefore, also δ^13^C of *f*_ci_ roughly equals δ^13^C of *A*. However, when *A* is low (e.g. mornings and evenings) the interpretation of the measured δ^13^C of *f*_ci_ becomes more difficult because of three factors:

(1) Changes in the carbon pools *ζ*_i_ and *ζ*_c_ (CO_2_ in intercellular airspaces and chloroplasts) break the equality between the fluxes of the steady‐state assumption ($f_{c,i}=f_{c,c}=A‐R_{d}‐R_{p}$).

(2) Deriving δ^13^C of *A* from the measured δ^13^C of *f*_ci_ requires accurate estimates of the rates and isotopic composition of *R*_d_ and *R*_p_. The significance of the accuracy of these estimates increases as the ratio $\frac{R_{d}+R_{p}}{A}$ increases.

(3) CO_2_ released in respiration enters some point within the path between ambient air and chloroplasts and thus, faces further discrimination on its way either to chloroplasts for refixation or to atmosphere, depending on the ratios between *C*_a_, *C*_i_ and *C*_c_.

The nonsteady‐state structure of the model allows us to evaluate the importance of these three factors for deriving δ^13^C of *A* from δ^13^C of *f*_ci_. To do that, we simulated the model with five assumptions (Table 3). We calculated the difference between the simulated δ^13^C of *A* and *f*_ci_, (i.e. $\delta^{13}CA‐\delta^{13}Cf_{\mathrm{ci}}$). In addition, for *f*_ci_ and δ^13^C *f*_ci_ we calculated the difference between the steady‐state value (i.e. value after the system reached steady state during the 15 min period) and the average value of the whole 15 min period.

**Table 3:** Tests for comparing parameterisation or model structure on the difference between $\delta^{13}C$ of *A* and *f*_ci_.

Case	Test	Changed parameter or formula
a	Standard	
b	The effect of pool volume on δ^13^C *A* and *f*_ci_	*V_i_*: 2e^−4^, *V_c_*: 6e^−4^, Fig. 2 and Eqn 1
c, d	The effect of respiration discrimination on δ^13^C *A* and *f*_ci_	‘high e and f’: *e* = −1, *f* = 16, ‘low e and f’: *e* = −11, *f* = 6 Ratios $R_{d}^{13}:R_{d}^{12}$and $R_{p}^{13}:R_{p}^{12}$in Fig. 2; Eqns 12, Supporting Information Eqn S1.3
e	The effect of the release location of respired carbon on δ^13^C *A* and *f*_ci_	$R_{d}^{j}$ and $R_{p}^{j}$ to *ζ_i_* instead of *ζ_c_* in Fig. 2 $\frac{d\zeta_{i,l}^{j}}{dt}=f_{\mathrm{ci}}^{j}‐f_{\mathrm{cc}}^{j}+R_{d}^{j}+R_{P}^{j}$ (Eqn 2) $\frac{d\zeta_{c,l}^{j}}{\mathrm{dt}}=f_{\mathrm{cc}}^{j}‐A^{j}$ (Eqn 3)

## Results

### Modelled and measured fluxes

**Fig. 3:**
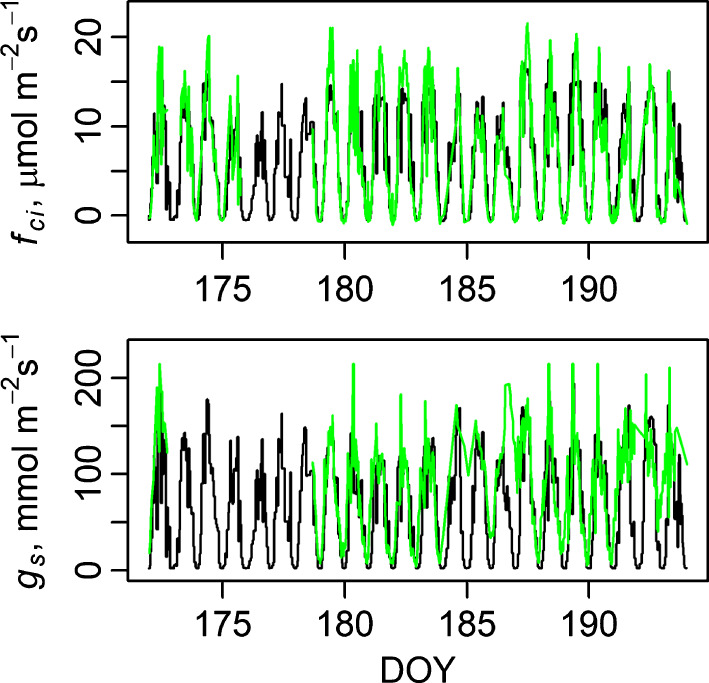
Measured (green) and modelled (black) *f*_ci_ (upper panel) and stomatal conductance (lower panel) of *Pinus sylvestris* during the days used for parameter estimation. Mesophyll conductance was modelled with *description 1* (Table 1). DOY, day of year.

Measured daily maxima in the carbon flux into the leaves (*f*_ci_) varied between 10 and 18 µmol m^−2^ s^−1^. Stomatal conductance to CO_2_ was close to zero during the night and 75–180 mmol m^−2^ s^−1^ at midday. The model was able to capture the measured variation in the carbon flux and *g*_s_ even though midday values were slightly underestimated (on average 4%) and some days showed clearly higher measured than simulated peak values (Fig. 3).

### Mesophyll conductance

**Fig. 4:**
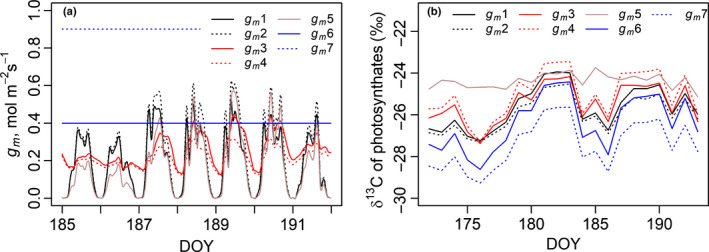
(a) Mesophyll conductance (mol m^−2^ s^−1^) of *Pinus sylvestris* during 7 d and (b) daily average isotopic composition of assimilated sugars during 22 d, modelled with *g_m_ descriptions 1–7* (Table 1). The driving factor of *g_m_* is *A/C_c_* in *descriptions 1* and *2*, temperature in *descriptions 3* and *4* and *A/C_c_* and temperature in *description 5*. A further reduction related to water stress is added to *descriptions 1* and *4*. *Descriptions 6* and *7* are constant *g*_m_. A shorter period is shown in (a) to more clearly visualise the within‐day patterns. DOY, day of year.

Different hypotheses regarding the driving factors of mesophyll conductance (*descriptions 1–7*; Table 1) resulted in varying daily patterns of *g*_m_. The average daytime maximum values of *descriptions 2*, *3*, *5* and *6* were parameterised to give c. 0.4 mol m^−2^ s^−1^ (Stangl *et al*., 2019) but the values varied among days depending on weather (Fig. 4a). *Descriptions 1*, *2* and *5* in which *g_m_* was driven by photosynthesis, showed night‐time values close to zero, whereas the temperature‐driven *descriptions* (*3* and *4*) showed a weaker diurnal cycle. The values of *g*_m_ with *descriptions* including a water‐stress reduction (*1* and *4*) were slightly lower than those of *descriptions* without a water‐stress effect (*2* and *3*, respectively).

*g*_m_ had a positive relationship with *g_s_* and net photosynthesis with *descriptions 1*, *2*, *3* and *5* either in a saturating (*1*), linear (*2* and *3*) or exponential (*5*) manner (Fig. S2a–d). The form of the relationship between net photosynthesis and *g*_m_/*g*_s_ resembled the positive saturating response found in the measurements of Stangl *et al*. (2019) in *descriptions 1* and *2* whereas the other *descriptions* led to an opposite form (Fig. S2e,f).

Differences in *g*_m_ resulted in differences in the daily average δ^13^C of the photosynthates (Fig. 4b). The differences in δ^13^C among *descriptions 1–6* (excluding *description* *7*, infinite *g*_m_) ranged from *c*. 1 per million to *c*. 3.5 per million, being largest between *descriptions 5* (photosynthetic rate and temperature as driving factors) and *6* (constant *g*_m_) under cold, cloudy conditions.

### Effect of different *g_m_* descriptions on phloem sugars

**Fig. 5:**
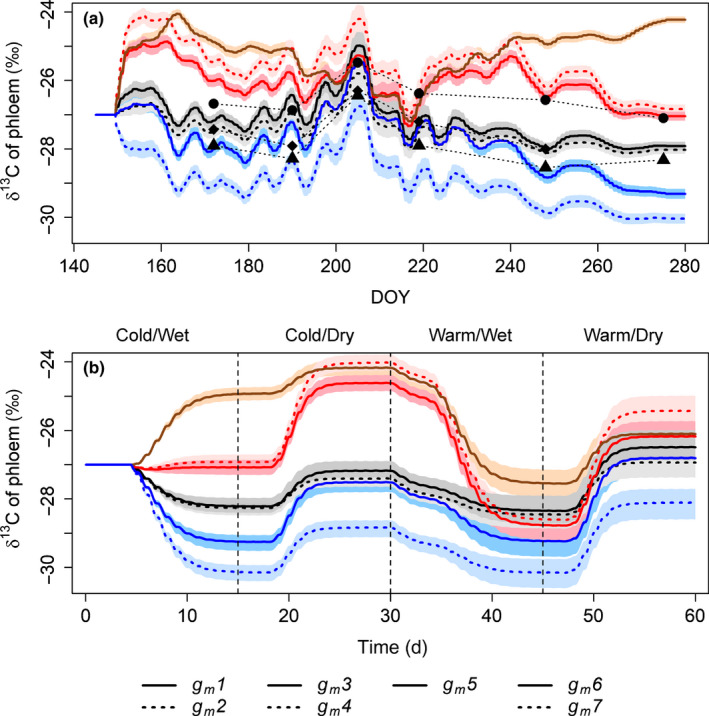
Simulated and measured isotopic composition of phloem sugars of *Pinus sylvestris* at breast height for: (a) year 2018 and (b) hypothetical, 60 d climate conditions with different mesophyll conductance descriptions (Table 1). The lines represent simulated values with ‘middle’ discrimination parameters *e* and *f* (*e* = −6, *f* = 11) whereas the shaded areas cover the ranges of ‘low’ and ‘high’ scenarios of parameters *e* and *f*, that is *e* = −11, *f* = 6 and *e* = −1, *f* = 16. Black symbols in (a) indicate measured phloem sugar δ^13^C values of three different trees. Vertical, dashed lines in (b) represent the timings of change in simulated hypothetical climate conditions.

The model was able to reproduce the strong drought‐related peak in the isotopic composition of phloem sugars detected during the summer in 2018, especially with *g*_m_ *descriptions 1*, *2*, *6* and *7* (Fig. 5a). Also the effect of precipitation in the end of July (DOYs 210 and 211) was visible in both the measured and modelled phloem δ^13^C. The overall level of phloem δ^13^C was the closest to the measured with *descriptions 1*, *2* and *6*.

With hypothetical environmental conditions (Fig. S1) cold days led to substantially larger discrepancies between the phloem δ^13^C among *g_m_ descriptions* than warm days (before and after day 30, respectively, Fig. 5b). Conversely, the differences between *g*_m_ *descriptions 1* and *2* vs low constant *g_m_* (*description* 6) were more pronounced during moist, low light conditions than during dry conditions. The time lag between environmental changes and the phloem δ^13^C reflected the rate of sugar transport from the foliage to the lower stem, whereas the small, direct temperature response (day 30) was caused by enhanced stem respiration.

Varying the values of *e* and *f*, that is discrimination in mitochondrial respiration and photorespiration, increased or decreased the δ^13^C of the photosynthates and thus phloem sugars (Fig. 5). The higher end of the range was reached during warm days with both mitochondrial and photorespiration being high.

### Within‐day variation of δ^13^C of assimilated sugars and parameter sensitivity

**Fig. 6:**
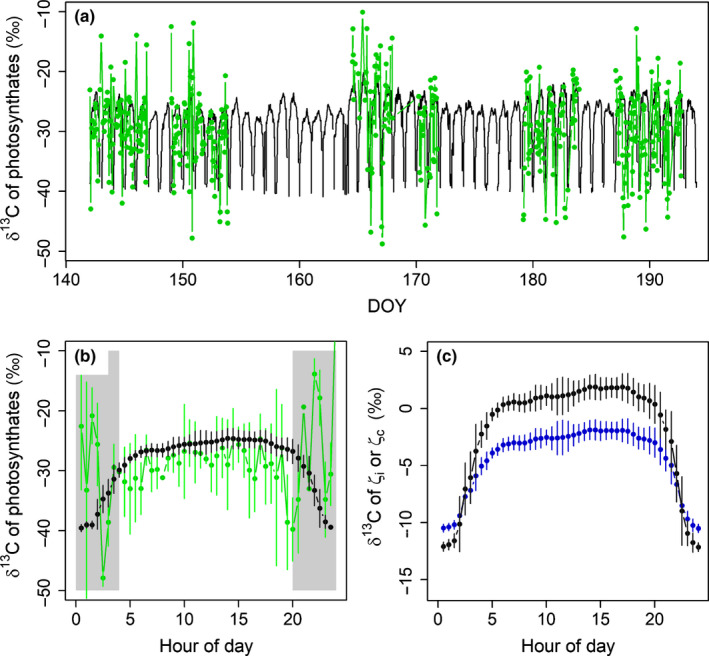
(a) Measured (green) and simulated (black) δ^13^C of the new photosynthates of *Pinus sylvestris* (b) Measured daily pattern of δ^13^C of *A*_net_ (green) and simulated daily pattern of δ^13^C of the new photosynthates (black). Both measured and simulated values are averaged over the measurement days. Standard deviation of measurements and model are shown with bars. The time periods at which uncertainty of the measurements is increased due to low light availability leading to low flux rates are indicated with grey panels. (c) Daily pattern of the simulated δ^13^C of the pools *ζ*_I_ (blue) and *ζ*_C_ (black) during the same days as in (a, b).

We used *g*_m_ *description 1* to study within‐day variation of the δ^13^C of assimilated sugars as it produced the closest correspondence with the measured pattern of *g*_m_/*g*_s_ vs *A*_net_ (Fig. S2) and with the measured pattern of phloem sugars in 2018 (Fig. 5a). The daily pattern of the simulated δ^13^C of the new photosynthates resembled the measured pattern (Stangl *et al*., 2019) between 05:00 and 20:00 h (Fig. 6a,b) being highest at noon and early afternoon. Modelled values decreased towards −40‰ close to midnight, while the measured values are inaccurate at low flux rates, that is early in the morning and late in the evening. δ^13^C in the pools *ζ*_I_ and *ζ*_C_ follow the same daily pattern with δ^13^C of *ζ*_C_ being on average 2.1‰ higher than δ^13^C of *ζ*_I_ (Fig. 6c).

Decreasing the value of *τ*_R_, the age of the mitochondrial respiration source carbon from 24 h to 5 h strengthened the diurnal pattern of the respiration δ^13^C (Fig. S3a). Changes in the direct temperature effect parameters only caused minor variation in the δ^13^C of photosynthates, at least during this mid‐summer period (Fig. S3b).

Increasing or decreasing the value of photosynthesis parameter α by 5 or 10%, while keeping the other parameters as estimated, resulted in a maximum 5 or 10% difference in photosynthesis, 5 or 11% difference in *g*_m_ and 0.09 or 0.18‰ difference in the δ^13^C of new photosynthates, depending on the *g_m_* description used (Fig. S4).

### Significance of nonsteady‐state assumption and respiration assumptions for deriving δ^13^C of photosynthates

**Fig. 7:**
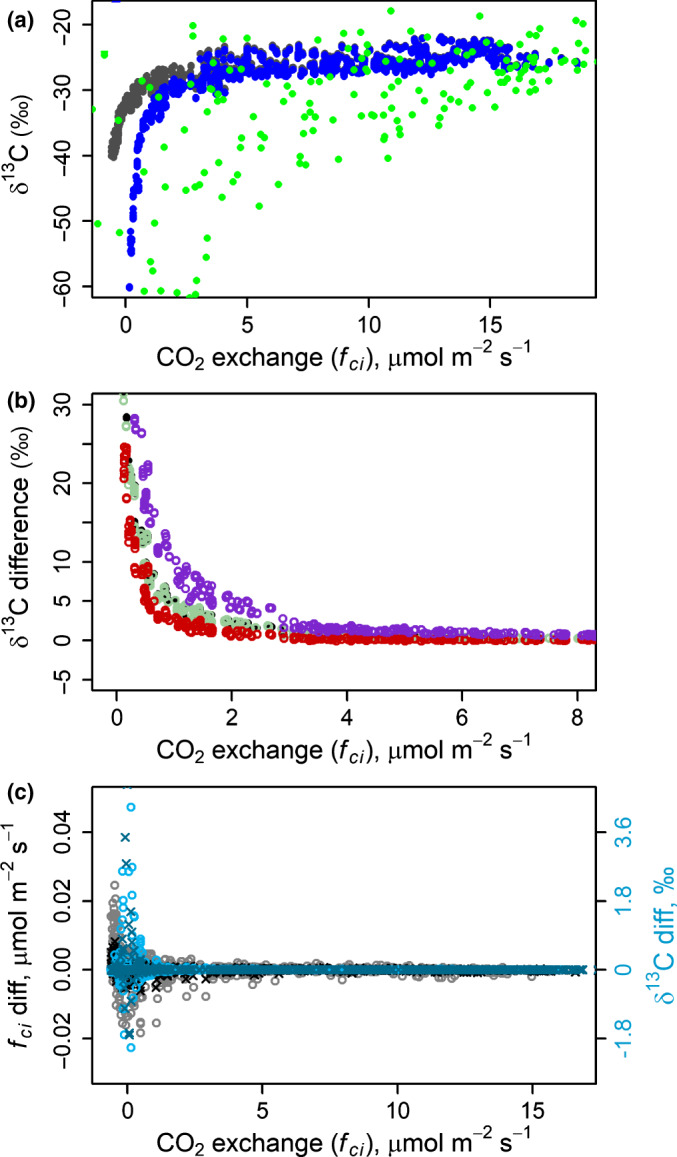
(a) δ^13^C of simulated photosynthesised sugars (*A*, black) and CO_2_ flux through stomata (*f*_ci_, blue) of *Pinus sylvestris*, average during each 15 min period. Green colour indicates δ^13^C of new photosynthates, estimated from cuvette measurements. (b) Difference between simulated δ^13^C of *A* and *f*_ci_ (black and blue dots in (a)). Black indicates results with standard parameterisation, green results with the assumption of respired carbon being released into the pool of *ζ_i_* instead of *ζ_c_*, purple results with respiration discriminations *e* = −11, *f* = 6 and red results with respiration discriminations *e* = −1, *f* = 16. (c) Difference between fluxes in the steady‐state situation (end of each 15 min simulation period) and average flux during the 15 min period, including the nonsteady‐state and the steady‐state period. Grey colours indicate CO_2_ flux (*f*_ci_) and blue colours δ^13^C of *f*_ci_. Crosses denote standard case parameters and circles denote results with increased *V_i_* and *V_c_*.

The δ^13^C of carbon flux into the leaf (*f*_ci_) was very close to δ^13^C of new photosynthates (*A*) when *f_ci _*> 3 µmol m^−2^ s^−1^ (Fig. 7a,b). However, when *f*_ci_ was small, that is *A* was close to *R*_d_ *+ P*_R_, the simulated δ^13^C of *A* was enriched compared with δ^13^C of *f*_ci_. The difference was larger with the assumption of ‘low *e* and *f*’, (*e* = −11, *f* = 6) but smaller with ‘high *e* and *f*’ (*e* = −1, *f* = 16). These effects were dominated by mitochondrial respiration discrimination. Changing the release location of respiratory CO_2_ only had a minor effect on the difference between δ^13^C of *A* and δ^13^C of *f*_ci_ compared with standard parameters (Fig. 7b), but led to an increase of δ^13^C of *c*. 0.4 per mil in the photosynthates.

The nonsteady‐state structure of the model was insignificant with *f_ci _*> 1 µmol m^−2^ s^−1^ (Fig. 7c). When *f_ci_* < 1 µmol m^−2^ s^−1^ both *f*_ci_ and δ^13^C *f*_ci_ calculated at the steady state (end of each 15 min simulation period) differed from the average value of the 15 min period. The effect was larger with larger volumes *V*_i_ and *V*_c_. The system reached steady state within 0–4 min with the standard parameterisation. The time for reaching steady state increased as flux decreased.

## Discussion

### Mesophyll conductance

We tested seven equations for describing *g*_m_, each based on previous published literature (Table 1). *Descriptions 1–5* connected *g*_m_ to photosynthetic rate, temperature and water stress whereas *descriptions 6* and *7* considered constant *g*_m_. Even though we set the average midday *g*_m_ in *descriptions 2*,*3*,*5* and *6* to correspond with the measured values reported by Stangl *et al*. (2019) the daily *g_m_* patterns, as well as daily average δ^13^C of photosynthates, varied due to differences in how the descriptions accounted for environmental variation (Fig. 4). Temperature affects *g_m_* through its physical effect on diffusion rate but also through processes requiring enzymes or other proteins (Bernacchi *et al*., 2002). *g*_m_ *descriptions* with a direct temperature dependence (*descriptions 3*, *4* and *5*) and those without (*descriptions 1*, *2*, *6* and *7*) led to different behaviours for *g_m_* and δ^13^C estimates between warm, sunny days and cold days (DOYs 190 and 186, respectively in Fig. 4). In addition, because temperature affects *A* it is included indirectly in all nonconstant *g*_m_ *descriptions* even though indirectly. Leaf water potential is suggested to affect *g_m_* either directly or by altering its temperature response (Li *et al*., 2020). Conversely, Shrestha *et al*. (2019) found no clear effect of water stress on the response of *g*_m_ to PPFD in chickpea. The water effect of our *descriptions 1* and *4* reduced *g_m_* in conditions of high VPD or low soil moisture (DOYs 188–190 in Fig. 4a).

In *descriptions 1* and *2*, adopted from Dewar *et al* (2018), *g*_m_ was proportional to *A/C*_c_ and thus, if *A* remained constant, *g*_m_ decreased when *C*_c_ increased. The equation was based on the optimisation of leaf photosynthesis under the assumption of nonstomatal constraints depending on leaf water status (Dewar *et al*, 2018). The nonstomatal constraints can be interpreted as *g*_m_ even though they do not provide a real mechanistic explanation. Interestingly, *descriptions 1* and *2* were the only ones that produced a similar pattern between net photosynthetic rate and *g*_m_/*g*_s_ measured by Stangl *et al* (2019). In fact, the other *descriptions* led to quite opposite patterns (Fig. S2). In accordance, Knauer *et al*. (2020) noted that most studies found a negative response of *g_m_* to *C_i_* and a positive response to light. While *descriptions 1* and *2* led to the closest correspondence with the measured *g*_m_/*g*_s_ vs *A*_net_ ratio, it must be borne in mind that these measurements only covered a few days with limited environmental variation. To provide a more stringent test between possible environmental responses of *g*_m_, the present method could be used in data sets covering a wider variety of weather conditions. In future model versions, it will also be possible to represent *g*_m_ in greater detail by including specific equations for diffusion through cell walls, plasmalemma, cytosol and chloroplast envelopes as for example Warren (2008) and Ubierna *et al*. (2019) suggested.

### Predicting phloem sugar isotopic composition from weather data

When phloem or tree ring isotopic data were used for backtracking past photosynthesis or water‐use efficiency, any explicit *g*_m_ estimate improves the obtained photosynthesis or WUE estimates compared with ignoring *g*_m_ (Sun *et al*., 2014). However, as discussed, *g*_m_ estimates may substantially differ under different weather conditions and different types of growing seasons (warm/dry vs cold/wet) may lead to substantially different average *g*_m_ depending on the description used. Thus, accurate inverse modelling requires an in‐depth understanding of the environmental effects on ^13^C discrimination. The present model was able to predict the drought‐related peak in phloem δ^13^C during summer 2018, especially with *g*_m_ *descriptions 1*, *2* and *6* (Fig. 5a), suggesting that the model was applicable to inverse modelling. Combining phloem δ^13^C data with weather and photosynthesis data allowed the quantification of the dependence of *g*_m_ on weather conditions (Ubierna & Marshall, 2011). Extreme weather events followed by a rapid change, provide the clearest signal for such analyses (Fig. 5). Here, the discrepancies of predicted phloem δ^13^C among *g*_m_ *descriptions* were largest during cold periods (Fig. 5b). Thus, at least for boreal Scots pines, we recommend short‐interval phloem sampling during and immediately after such periods, taking also into account the transportation time lag. Conversely, dry conditions that decrease photosynthesis seem to be suitable for distinguishing between the *descriptions* based on Dewar *et al* (2018) (*descriptions 1* and *2*) and constant *g_m_*, such as *description 6* (Fig. 5b) and thus choosing between the *descriptions* would benefit from frequent sampling during dry periods followed by rains. The model structure is applicable for other species as well, but species‐specific process parameters should obviously be changed. With other tree species, vertical transport of CO_2_ in the xylem is a potential process to be considered, even though it seems negligible in Scots pine (Tarvainen *et al*., 2020).

Studies reporting clear climate signals in δ^13^C of phloem sugars or tree rings indicated that the isotopic composition of photosynthates largely remains constant as they are transported from leaves to the sink tissues (Högberg *et al*., 2008; Rascher *et al*., 2010). However, it is also well known that isotopic discrimination related to postphotosynthetic processes, as well as mixing of newly assimilated carbon with older carbon pools, dampen the connection between δ^13^C of photosynthesised sugars and either nonstructural or structural carbon measured in sink tissues (Badeck *et al*., 2005; Gessler *et al*., 2009; Ogée *et al*., 2009; Rinne *et al*., 2015). Specifically, Tcherkez *et al*. (2004) found an effect of starch synthesis/breakdown on the isotopic composition of leaf sugars, and a ^13^C labelling experiment by Desalme *et al*. (2017) suggested that the mean residence time of newly assimilated carbon in pine needles was 1–3 d depending on the season. Such processes possibly altering the signal have to be accounted for to achieve correct predictions (Ogée *et al*., 2009; Zeng *et al*., 2017). Wingate *et al*. (2010) observed a 2–10 d delay and a dampening of the short‐term variation in the respiration δ^13^C signal when comparing photosynthetic isotope discrimination of *Pinus pinaster* with subsequent measurements of isotopic compositions of stem, soil and ecosystem respiration. Furthermore, significant variation in the δ^13^C among different sugar compounds of leaves and phloem sap has been reported (Merchant *et al*, 2011; Rinne *et al*., 2015). The present model only accounts for discrimination related to respiration along the pathway from leaves towards roots. Respired CO_2_ is usually enriched compared with the substrate (Duranceau *et al*., 2001; Ghashghaie *et al*., 2001). Werner & Gessler (2011) and Lehmann *et al*. (2016) observed respired carbon to be heaviest during early afternoon and was in agreement with our model results, although the daily variation (up to 6‰, Werner & Gessler, 2011) in the previous observations is more pronounced than in our simulation (up to 3‰; Fig. S3a). Discrimination related to transport, growth and conversion processes can easily be adopted into the model when knowledge about these processes accumulates. In the current state of the model, the assumptions related to for example proportions of sugars transported downwards from different canopy layers are very simplified. Although we think these assumptions are reasonable and thus do not expect a very large impact on model results for this analysis, it would be possible to replace the simple description of sugar transport with a mechanistic transportation and growth carbon sink model, such as presented by Hölttä *et al*. (2017), and/or modify the canopy model by increasing the number of canopy layers, separating sun and shade leaves or considering light attenuation within the canopy. These changes would probably make the model more accurate in predicting variations in δ^13^C at a finer scale than the extreme drought release effect in 2018. Verifying the whole tree model would also benefit from more detailed δ^13^C measurements along the transport path, including at least some of the following compartments: leaf nonstructural and structural carbon as well as branch and stem phloem and xylem nonstructural and structural carbon.

### δ^13^C of recent photosynthates: within‐day variation and the significance of nonsteady‐state respiration assumptions

The simulated photosynthates were most enriched during midday (Fig. 6). The simulated pattern followed the measurements between 04:00 and 20:00 h, but the connection broke down outside this period as the measured δ^13^C increased, whereas the simulated values decreased (Fig. 6b). Uncertainties in the measured δ^13^C of *f_ci_* increased as CO_2_ flux decreased (Pons *et al*., 2009; Stangl *et al*., 2019). Furthermore, the inference of δ^13^C of new sugars includes assumptions about the values of parameters *e* and *f* and about refixation of respired CO_2_. This was also noted by Bickford *et al*. (2010) who did not succeed in predicting diurnal variation in larch ^13^C discrimination. They interpreted this to emphasise the effect of unaccountable factors related to, for example, *g_m_* or fractionation of respiration. Indeed, determining the correct early morning and late evening δ^13^C remains challenging. The responses of the δ^13^C of recent photosynthates to varying respiration parameters or assumptions are however able to be studied by modelling.

The validity of the original isotopic discrimination model by Farquhar *et al*. (1982), at low photosynthetic rates, was recently challenged by Busch *et al*. (2020). They modified the model assumptions related to mitochondrial respiration, compared the new model with measured *g_m_* values and found that the new model performed better than the original when *R*/*A* was large. Following those results, we evaluated here at which flux rates the discrepancy between the δ^13^C of *f*_ci_ and photosynthates, or the discrepancy between steady‐state and nonsteady‐state *f*_ci_ or δ^13^C *f*_ci_, increased. With all our tests, δ^13^C of *A* equalled δ^13^C of *f*_ci_ when *f*_ci_ > 3 µmol m^−2^ s^−1^ and steady‐state *f*_ci_ and δ^13^C *f*_ci_ equalled their nonsteady‐state values when *f*_ci_ > 1 µmol m^−2^ s^−1^ (Fig. 7), that is *A *>> *R*. Obviously, most of the photosynthates were produced during high *A* and under such conditions assumptions related to: (1) carbon pool sizes, (2) respiration parameters, or (3) the release location of respired carbon did not have an effect on the inference of δ^13^C of *A* from the δ^13^C of *f*_ci_. However, understanding the within‐day variation of δ^13^C requires quantification of the responses of the system to these assumptions at low flux. In line also with the results of Ubierna *et al*. (2019), the assumptions began to play a role with *f*_ci_ = 0.5–3 µmol m^−2^ s^−1^ and their effect rapidly increased as *f_ci_* approached zero, that is *A/R* approached one, especially with strong mitochondrial respiration discrimination. The volumes *V*_i_ and *V*_c_ affected the results after changes in the weather, when the changes in the pool sizes acted as a buffer between fluxes (Fig. 7c). Real environmental variability, especially light, is much faster than 15 min and this may lead to somewhat different mean values than assuming a mean environment for example of 15 min. The larger the volumes, the slower the steady state is reached and the larger is the effect. Thus, thick leaves and high‐frequency environmental input increased the relevance of the nonsteady‐state assumption, especially when studying phenomena related to morning or evening times.

### Conclusions

We developed a dynamic model to predict isotopic signatures of photosynthates and phloem sugars based on different assumptions of *g*_m_ responses to environment, and compared the results with measured data. The model resulted in different δ^13^C of new photosynthates with different *g*_m_ descriptions. Our results showed that *g*_m_ *description 1* determined by the photosynthetic rate, CO_2_ concentration in chloroplasts and water availability yielded the closest agreement with observations during the studied mid‐summer period. We note, however, that this result remains to be confirmed with data sets collected under more varying environmental conditions. The model succeeded in predicting the drought responses of year 2018 phloem sugars, which indicates the possibility of using the model for backtracking *g*_m_ with tree ring isotopic and weather data.

## Author contributions

PS‐A, AM, ZRS and JM planned the study. PS‐A and AM constructed the model. ZRS, LT and GW conducted the measurements. PS‐A conducted model analysis, all authors contributed to planning the analyses. ZRS conducted the measurement data analysis. PS‐A was responsible for writing the manuscript. All authors contributed to the writing of the manuscript at various stages.

## Supporting information


**Fig. S1** Hypothetical environmental conditions used in analysis.
**Fig. S2** The relationships between *g*_m_ and *g*_s_, *g*_m_ and *A*_net_, and *g*_m_ */g*_s_ and *A*_net_ based on seven different *g*_m_ *descriptions*.
**Fig. S3** The effects of *τ*_R_ and direct temperature response parameters on model results.
**Fig. S4** Sensitivity of model results to photosynthesis parameter α.
**Methods S1** Other model variables.
**Methods S2** Parameter sensitivity.
Please note: Wiley Blackwell are not responsible for the content or functionality of any Supporting Information supplied by the authors. Any queries (other than missing material) should be directed to the *New Phytologist* Central Office.

## Data Availability

Data are available on request from the authors.
