# Supplementary material for: Linking canopy‐scale mesophyll conductance and phloem sugar δ13C using empirical and modelling approaches
Source: New Phytol. 2020 Dec 19;229(6):3141–55. doi: 10.1111/nph.17094 (PMC7986199; doi:10.1111/nph.17094)
Supplement: Supplementary file 1 — Fig. S1 Hypothetical environmental conditions used in analysis. Fig. S2 The relationships between g m and g s, g m and A net, and g m /g s and A net based on seven different g m descriptions. Fig. S3 The effects of τ R and direct temperature response parameters on model results. Fig. S4 Sensitivity of model results to photosynthesis parameter α. Methods S1 Other model variables. Methods S2 Parameter sensitivity. Please note: Wiley Blackwell are not responsible for the content or functionality of any Supporting Information supplied by the authors. Any queries (other than missing material) should be directed to the New Phytologist Central Office. [file NPH-229-3141-s001.pdf]

New Phytologist

New Phytologist Supporting Information

Article title: Linking canopy-scale mesophyll conductance and phloem sugar $\delta^{13}C$ using empirical and modelling approaches

Authors: Pauliina Schiestl-Aalto, Zsofia R.Stangl, Lasse Tarvainen, Göran Wallin, John Marshall, Annikki Mäkelä

Article acceptance date: 16 November 2020

The following Supporting Information is available for this article:

Methods S1 Other model variables

Other variables needed for the flux calculations are determined as follows:

Stomatal conductance (mol m^{-2} s^{-1}) was modelled with Ball–Berry–Leuning function (Leuning 1995) as

$$g_s(t) = g_s^0 + \frac{a_1 A^{12}(t)}{(C_a(t) - \Gamma^*(t))(1+\frac{D(t)}{D_0})} \quad (S1.1)$$

where g_s^0 (mol m^{-2} s^{-1}) is the minimum stomatal conductance, C_a is ambient CO_2 concentration (mol mol^{-1}), D is VPD (kPa) and a_1 and D_0 are empirical parameters.

Mitochondrial respiration rate is calculated following Launiainen *et al.* (2015) as

$$R_d^{12}(t) = l_e R_{d,25} e^{r_1(T(t)-T_{N25}) / r_2 R T(t)} \quad (S1.2)$$

where $R_{d,25}$ is mitochondrial respiration rate in 25 °C temperature, $T(t)$ is air temperature (K), T_{N25} is 298.15 K, R is the molar gas constant and l_e represents the reduction of mitochondrial respiration in light, r_1 and r_2 are parameters and

$$R_d^{13}(t) = R_s(t) \frac{R_d^{12}(t)}{1+e} \quad (S1.3)$$

where R_s is the isotopic ratio of carbon in needle sugar pool ($\frac{\zeta_s^{13}}{\zeta_s^{12}}$) and e is a ^{13}C discrimination parameter related to photorespiration (Ghashghaie *et al.* 2003).

Mitochondrial respiration decreases with light (Tcherkez *et al.* 2017). Thus, variable l_e (unitless) restricts mitochondrial respiration during day time.

$$l_e(t) = \begin{cases} 1, & I(t) < I_{RT} \\ 0.6, & I(t) \geq I_{RT} \end{cases} \qquad \text{(S1.4)}$$

where I is PAR (mol m^{-2} s^{-1}) and I_{RT} threshold PAR for respiration restriction.

The light compensation point of photosynthesis Γ^* (mol mol^{-1}) is modelled following Medlyn *et al.* (1999) as

$$\Gamma^*(t) = g_1 + g_2(T(t) - T_{N25}) + g_3(T(t) - T_{N25})^2 \qquad \text{(S1.5)}$$

where $T(t)$ is ambient temperature (K), g_1, g_2 and g_3 are coefficients.

The direct effect of temperature of photosynthetic rate is calculated as

$$f_T^A(t) = \frac{1}{1+e^{-d_1(T_C(t)-d_2)}} \qquad \text{(S1.6)}$$

where $T_c(t)$ is temperature and d_1 and d_2 are coefficients. Following Mäkelä *et al.* (2008) the lagged effect of temperature describing seasonality ($S(t)$) is calculated as

$$f_T^S(t) = min\left\{\frac{S(t)}{S_{max}}, 1\right\} \qquad \text{(S1.7)}$$

where S_{max} (°C) is the threshold where temperature acclimation reaches its maximum and

$$\frac{dS(t)}{dt} = \frac{T_c(t)-S(t)}{\tau_S} \quad \text{(S1.8)}$$

where $T_c(t)$ is temperature (°C) and τ_s a time delay parameter.

The reduction of mesophyll conductance in *descriptions 1* and *4* caused by water stress is calculated as

$$r_W(t) = 1 - r_D(t) - r_S(t) \quad \text{(S1.9)}$$

where reduction caused by VPD (r_D) is determined as

$$r_D(t) = \frac{D}{p_D} \quad \text{(S1.10)}$$

where p_D is a parameter. Reduction caused by soil moisture (r_S) is derived following Peltoniemi *et al.* (2015)

$$r_S(t) = \frac{1-W(t)}{p_S} \quad \text{(S1.11)}$$

where $W(t)$ is relative extractable water and p_S is a parameter.

$$W(t) = \frac{\theta(t)-\theta_{WP}}{\theta_{FC}-\theta_{WP}} \quad \text{(S1.12)}$$

Where $\theta(t)$ is soil moisture (m^3 m^{-3}), θ_{WP} is wilting point (m^3 m^{-3}) and θ_{FC} field capacity.

The reduction of mesophyll conductance in *descriptions 3-5* caused by temperature following Sun *et al.* (2014):

$$r_T(t) = \frac{e^{p_1-p_2/(RT(t))}}{1+e^{(p_3T(t)-p_4)/(RT(t))}} \quad \text{(S1.13)}$$

where p_1, p_2, p_3 and p_4 are coefficients.

The reduction of mesophyll conductance in *descriptions 4* and *5* caused by light environment in different parts of the canopy is expressed as:

$$r_I^l = \begin{cases} i_I^1, \text{ for top part of the canopy} \\ i_I^2, \text{ for middle part of the canopy} \\ i_I^3, \text{ for lowest part of the canopy} \end{cases} \quad \text{(S1.14)}$$

where i_I^1, i_I^2 and i_I^3 are parameters visually estimated from Sun *et al.* (2014).

References:

Ghashghaie J, Badeck FW, Lanigan G, Nogués S, Tcherkez G, Deléens E, Cornic G, Griffiths H. 2003. Carbon isotope fractionation during dark respiration and photorespiration in C_3 plants. *Phytochemistry Reviews* **2**: 145-161. doi: 10.1023/B:PHYT.0000004326.00711.ca

Launiainen S, Katul GG, Lauren A, Kolari P. 2015. Coupling boreal forest CO_2, H_2O and energy flows by a vertically structured forest canopy – soil model with separate bryophyte layer. *Ecological modelling* **312:** 385-405. doi: 10.1016/j.ecolmodel.2015.06.007

Leuning R. 1995. A critical appraisal of a combined stomatal-photosynthesis model for C_3 plants. *Plant, Cell and Environment* **18:** 339–355. doi: 10.1111/j.1365-3040.1995.tb00370.x

Mäkelä A, Pulkkinen M, Kolari P, Lagergren F, Berbigier P, Lindroth A, Loustau D, Nikinmaa E, Vesala T, Hari P. 2008. Developing an empirical model of stand GPP with the LUE approach: analysis of eddy covariance data at five contrasting conifer sites in Europe. *Global Change Biology* **14:** 92–108. doi: 10.1111/j.1365-2486.2007.01463.x

Medlyn BE, Badeck FW, Pury DGGD, Barton CVM, Broadmeadow M, Ceulemans R, Angelis PD, Forstreuter M, Jach ME, Kellomäki S, et al. 1999. Effects of elevated CO_2 on photosynthesis in European forest species: a meta-analysis of model parameters. *Plant, Cell and Environment* **22 (12):** 1475–1495. doi: 10.1046/j.1365-3040.1999.00523.x

Peltoniemi M, Pulkkinen M, Aurela M, Pumpanen J, Kolari P, Mäkelä A. 2015. A semi-empirical model of boreal-forest gross primary production, evapotranspiration, and soil water - calibration and sensitivity analysis. *Boreal Environment Research* **20:** 151–171.

Sun Y, Gu L, Dickinson RE, Norby RJ, Pallardy SG, Hoffman FM. 2014. Impact of mesophyll diffusion on estimated global land CO_2 fertilization. *Proceedings of the National Academy of Sciences* 111 **(44):** 15774-15779. doi: 10.1073/pnas.1418075111

New Phytologist

Tcherkez G, Gauthier P, Buckley TN, Busch FA, Barbour MM, Bruhn D, Heskel MA, Gong XY, Crous KY, Griffin K, et al. 2017. Leaf day respiration: low CO_2 flux but high significance for metabolism and carbon balance. *New Phytologist* **216:** 986-1001. doi:10.1111/nph.14816

Methods S2 Parameter sensitivity

To test the effect of parameters *e* and *f*, i.e. discrimination of mitochondrial respiration and photorespiration (eqs S1.3 and 12) we used parameter combinations "low": *e* = -11, *f* = 6, "middle": *e* = -6, *f* = 11 and "high": *e* = -1, *f* = 16, to see how the phloem sugar δ^{13}C results respond to low or high respiration discrimination. The values were chosen to cover the upper and lower limits of the suggested values of *e* and *f* for several species (Ghashghaie *et al.* 2003, Cernusak *et al.* 2013, Ubierna *et al.* 2019).

Pool ζ_s (Fig. **2**) is the source pool of carbon for mitochondrial respiration. We set the time constant of the carbon residence time in ζ_s (parameter τ_R, eq. 14) to 24 hours which means that the carbon used for respiration is a mixture of carbon that was photosynthesized during ca. the previous day. To test the effect of this value on the δ^{13}C of respired carbon we changed the value to 5 hours.

In the photosynthesis model, low temperature directly decreases the photosynthetic rate (eq. 9). We varied the parameters d_1 and d_2 of eq. S1.6 to test how steeper or flatter temperature responses affect the within day or among days δ^{13}C results. For this, we used combinations of parameters d_1 and d_2 of 0.11 and 5 vs. 0.06 and -17 for steeper and flatter response curve, respectively. For reference values of d_1 and d_2 (0.08 and -5), photosynthetic rate at 10 °C is decreased by 24 % compared with maximum reached at 35 °C. Using the steeper and flatter curves resulted in reductions by 37 % and 17 %, respectively.

Furthermore, we tested the sensitivity of the simulated photosynthesis rate, mesophyll conductance and daily average δ^{13}C of new photosynthates on varying parameter α while keeping other parameters as estimated. We ran the model with each g_m description with α increased or decreased by 5 % or 10 % of the original, g_m *description* specific α, estimated as explained in section 2.5.7. We calculated mean and maximum differences between the model results with changed and original α over a 22 day period. For photosynthesis rate and mesophyll conductance, we calculated a relative difference, e.g. $g_{m,test}$ / $g_{m,original}$ whereas for δ^{13}C of new photosynthates, we calculated the absolute difference, $\delta^{13}C_{test}$ - $\delta^{13}C_{original}$.

References:

Cernusak LA, Ubierna N, Winter K, Holtum JAM, Marshall JD, Farquhar GD. 2013. Environmental and physiological determinants of carbon isotope discrimination in terrestrial plants. *New Phytologist* **200:** 950–965. doi: 10.1111/nph.12423

Ghashghaie J, Badeck FW, Lanigan G, Nogués S, Tcherkez G, Deléens E, Cornic G, Griffiths H. 2003. Carbon isotope fractionation during dark respiration and photorespiration in C_3 plants. *Phytochemistry Reviews* **2:** 145-161. doi: 10.1023/B:PHYT.0000004326.00711.ca

Ubierna N, Cernusak LA, Holloway-Phillips M, Busch FA, Cousins AB, Farquhar GD. 2019. Critical review: incorporating the arrangement of mitochondria and chloroplasts into models of photosynthesis and carbon isotope discrimination. *Photosynthesis Research* **141:** 5-31. doi: 10.1007/s11120-019-00635-8

Fig. S1 Hypothetical environmental conditions used in analysis

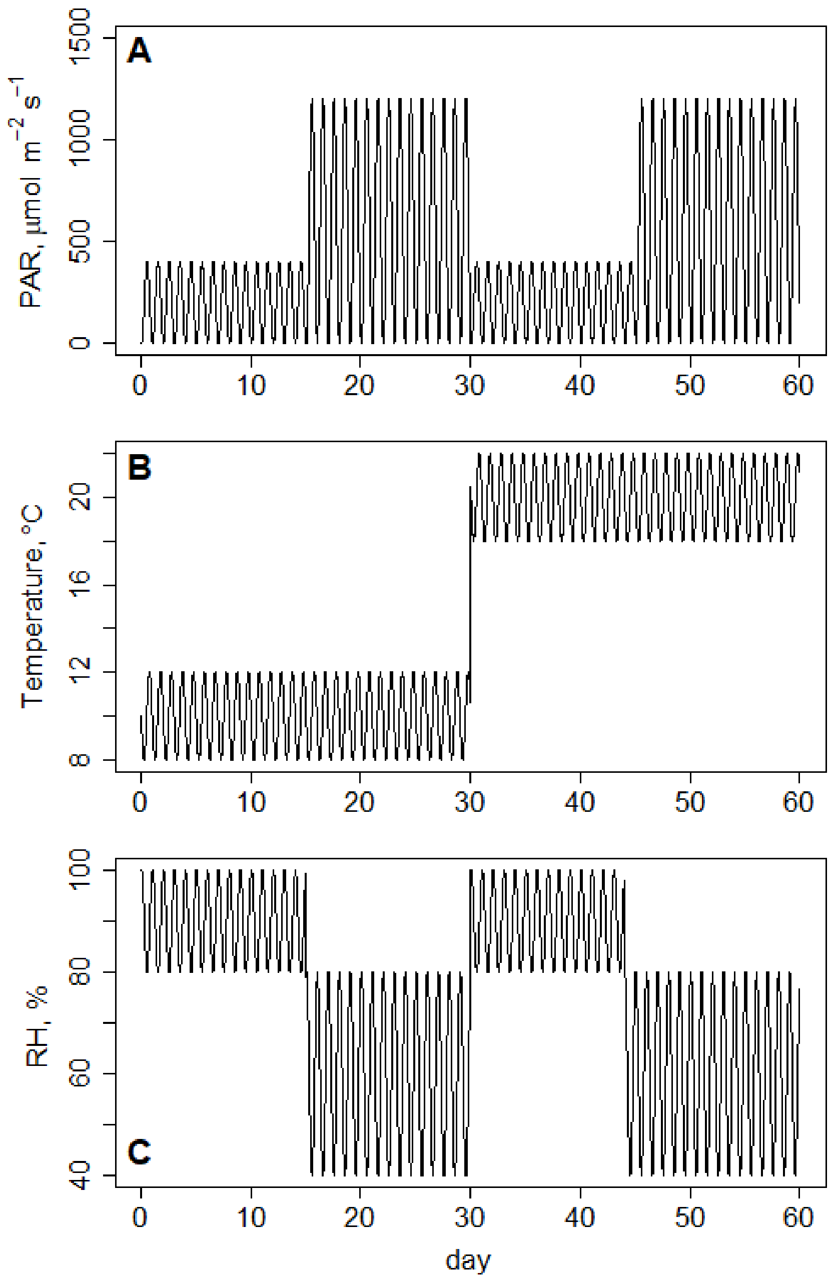

Fig. S1. Hypothetical environmental conditions used in analysis of the effects of environmental conditions on $\delta^{13}C$ of phloem sugars with different mesophyll conductance equations. A: photosynthetically active radiation (PAR), B: air temperature, C: relative humidity (RH).

Fig. S2 The relationships between g_m and g_s, g_m and A_{net}, and g_m/g_s and A_{net} based on seven different g_m *descriptions*.

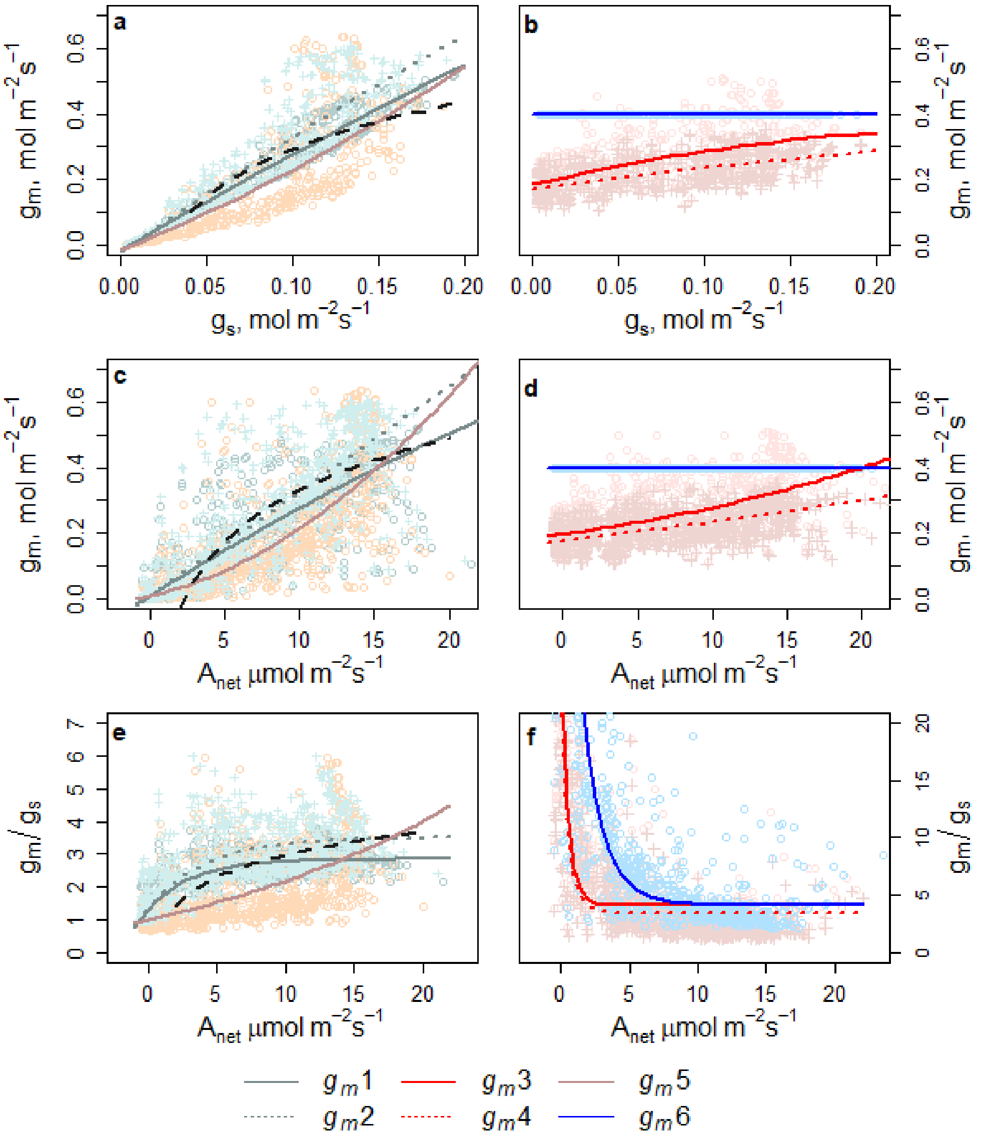

Fig. S2. The relationships between g_m and g_s (figs a, b), g_m and A_{net} (figs c, d) and g_m/g_s and A_{net} (figs e, f) based on seven different g_m *descriptions* (g_m*1-6*, Table 1). *Descriptions 1, 2* and *5* that are driven by A/C_c are shown on left-hand side (figs a, c, e) and *descriptions 3, 4* and *6* on right-hand side (figs b, d, f). Dots are the model results and smoothed lines are added to show the patterns. Black dashed lines in panels a, c and e represent measured estimates. Note the different y-axis in e and f.

Fig. S3 The effects of τ_R and direct temperature response parameters on model results.

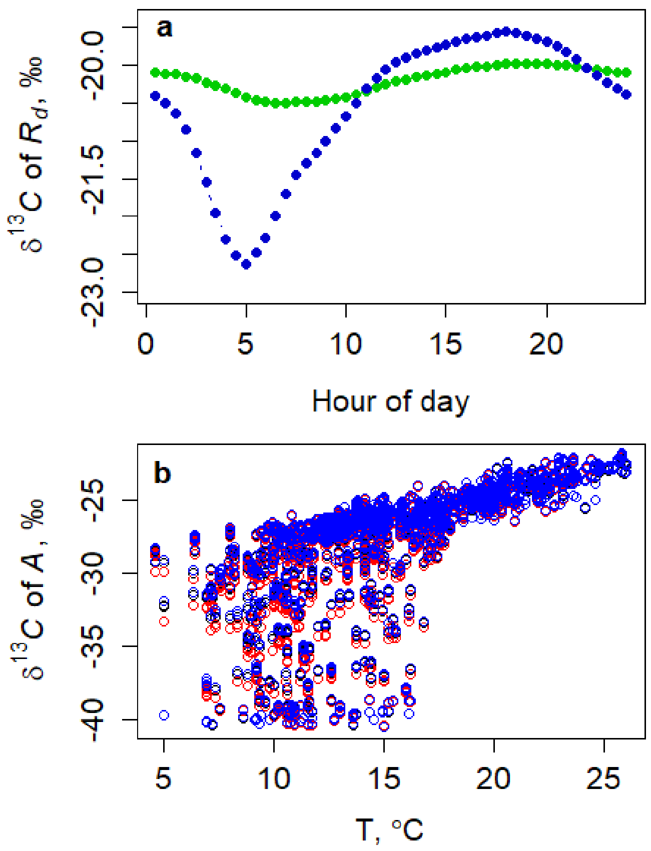

Fig. S3. a) The effect of τ_R, i.e. the average age of the sugars that are used for mitochondrial respiration on the daily pattern of δ^{13}C of respiration. Green colour indicates mixing time of one day, blue colour mixing time of five hours. b) δ^{13}C of modelled photosynthesized sugars with different direct temperature response parameters of photosynthesis. Black dots represent the original parameter values, blue dots flatter temperature response and red dots steeper temperature response.

Fig. S4 Sensitivity of model results to photosynthesis parameter α.

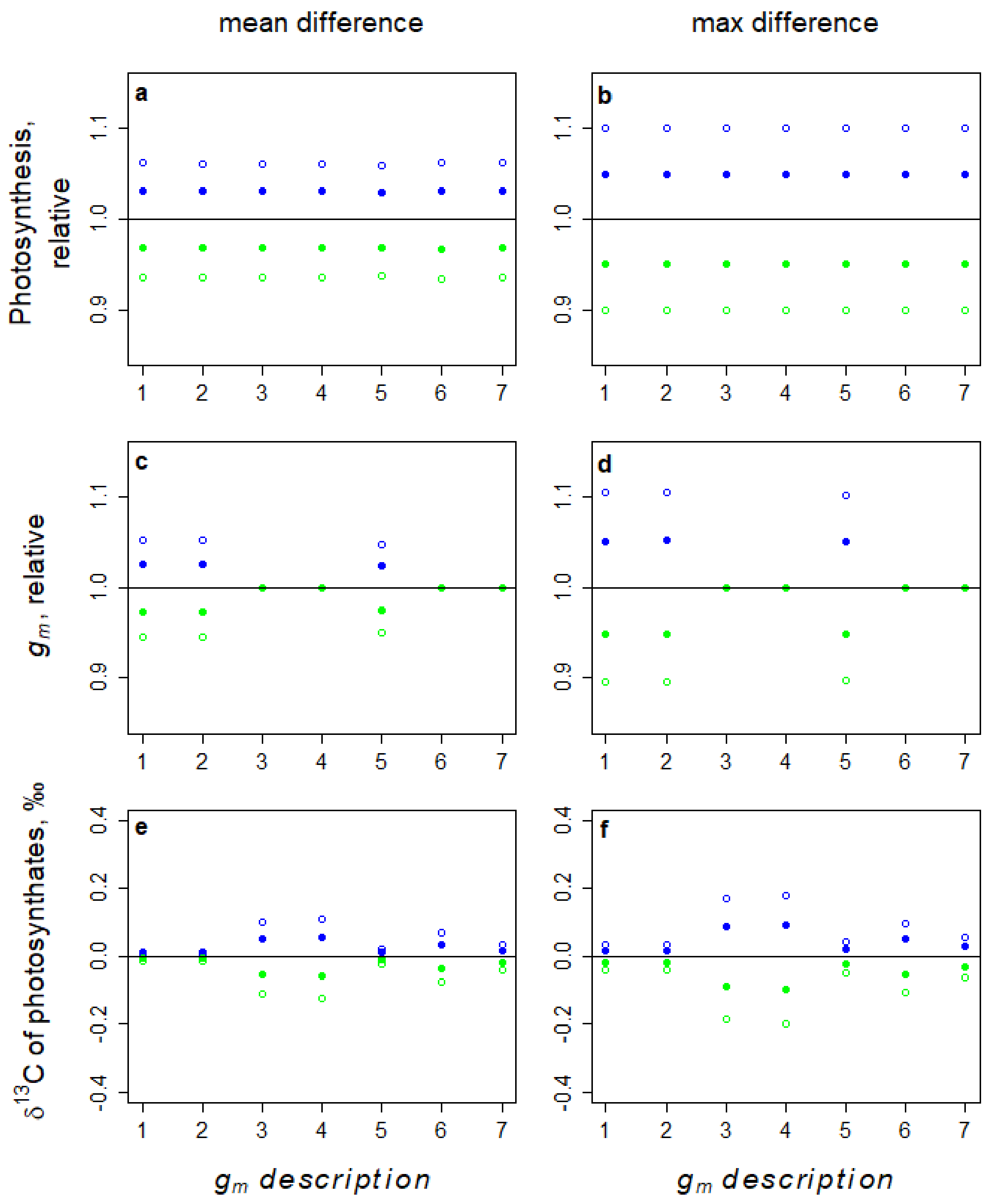

Fig S4. The effect of changing the value of photosynthesis parameter α on photosynthesis rate (a, b), mesophyll conductance (c, d) and daily average $\delta^{13}C$ of new photosynthates with the seven different g_m *descriptions*. α was increased or decreased by 10 % (blue and green open circles, respectively) and 5 % (blue and green filled circles, respectively) and model results were compared to those with α value estimated as explained in section 2.5.7. Panels a-d show the relative difference, e.g. $g_{m,test}$ / $g_{m,original}$ whereas panels e-f show the absolute difference, $\delta^{13}C_{test}$ -

$\delta^{13}C_{original}$. Panels a, c, and e show the mean differences and panels b, d and f the maximum differences over a 22 day period.
